# Neutrophil‐to‐lymphocyte ratio and outcomes in patients with new‐onset or worsening heart failure with reduced and preserved ejection fraction

**DOI:** 10.1002/ehf2.13424

**Published:** 2021-05-16

**Authors:** Fraser M. Curran, U Bhalraam, Mohapradeep Mohan, Jagdeep S. Singh, Stefan D. Anker, Kenneth Dickstein, Alexander S. Doney, Gerasimos Filippatos, Jacob George, Marco Metra, Leong L. Ng, Colin N. Palmer, Nilesh J. Samani, Dirk J. van Veldhuisen, Adriaan A. Voors, Chim C. Lang, Ify R. Mordi

**Affiliations:** ^1^ School of Medicine University of Dundee Dundee UK; ^2^ Division of Molecular and Clinical Medicine University of Dundee Dundee UK; ^3^ Department of Cardiology (CVK); and Berlin Institute of Health Center for Regenerative Therapies (BCRT); German Centre for Cardiovascular Research (DZHK) partner site Berlin Charité Universitätsmedizin Berlin Berlin Germany; ^4^ University of Bergen Stavanger University Hospital Stavanger Norway; ^5^ Division of Population Health and Genomics University of Dundee Dundee UK; ^6^ Heart Failure Unit, Department of Cardiology, School of Medicine National and Kopodistrian University of Athens, Athens University Hospital Attikon Athens Greece; ^7^ Institute of Cardiology, Department of Medical and Surgical Specialties, Radiological Sciences and Public Health University of Brescia Brescia Italy; ^8^ Department of Cardiovascular Sciences University of Leicester and NIHR Leicester Biomedical Research Centre Leicester UK; ^9^ Department of Cardiology, University Medical Center Groningen University of Groningen Groningen The Netherlands

**Keywords:** Heart failure, Neutrophil‐to‐lymphocyte ratio, Inflammation, Biomarkers, Outcome

## Abstract

**Aims:**

Inflammation is thought to play a role in heart failure (HF) pathophysiology. Neutrophil‐to‐lymphocyte ratio (NLR) is a simple, routinely available measure of inflammation. Its relationship with other inflammatory biomarkers and its association with clinical outcomes in addition to other risk markers have not been comprehensively evaluated in HF patients.

**Methods:**

We evaluated patients with worsening or new‐onset HF from the BIOlogy Study to Tailored Treatment in Chronic Heart Failure (BIOSTAT‐CHF) study who had available NLR at baseline. The primary outcome was time to all‐cause mortality or HF hospitalization. Outcomes were validated in a separate HF population.

**Results:**

1622 patients were evaluated (including 523 ventricular ejection fraction [LVEF] < 40% and 662 LVEF ≥ 40%). NLR was significantly correlated with biomarkers related to inflammation as well as NT‐proBNP. NLR was significantly associated with the primary outcome in patients irrespective of LVEF (hazard ratio [HR] 1.18 per standard deviation increase; 95% confidence interval [CI] 1.11–1.26, *P* < 0.001). Patients with NLR in the highest tertile had significantly worse outcome than those in the lowest independent of LVEF (<40%: HR 2.75; 95% CI 1.84–4.09, *P* < 0.001; LVEF ≥ 40%: HR 1.51; 95% CI 1.05–2.16, *P* = 0.026). When NLR was added to the BIOSTAT‐CHF risk score, there were improvements in integrated discrimination index (IDI) and net reclassification index (NRI) for occurrence of the primary outcome (IDI + 0.009; 95% CI 0.00–0.019, *P* = 0.030; continuous NRI + 0.112, 95% CI 0.012–0.176, *P* = 0.040). Elevated NLR was similarly associated with adverse outcome in the validation cohort. Decrease in NLR at 6 months was associated with reduced incidence of the primary outcome (HR 0.75; 95% CI 0.57–0.98, *P* = 0.036).

**Conclusions:**

Elevated NLR is significantly associated with elevated markers of inflammation in HF patients and is associated with worse outcome. Elevated NLR might potentially be useful in identifying high‐risk HF patients and may represent a treatment target.

## Introduction

There has been long‐standing interest in the role of inflammation in the pathophysiology of heart failure (HF).^1^ It has been shown that both heart with reduced ejection fraction (HFrEF) and heart with preserved ejection fraction (HFpEF) are associated with elevations in biomarkers related to inflammatory processes, although the exact mechanisms by which inflammation is thought to contribute towards incidence and prognosis of HF are not fully understood.^2^, ^3^ It is hypothesized that the immune system has a key role in initiating an inflammatory response that is, at least in part, cardioprotective in the short term, but, if sustained, can lead to a state of chronic low‐grade inflammation, which may accelerate disease progression.^2^ Several studies have highlighted the association between raised inflammatory biomarkers and adverse outcomes in HF.^3^, ^4^, ^5^, ^6^, ^7^, ^8^ However, none of these biomarkers have been translated to measurement in routine clinical practice.

Neutrophil‐to‐lymphocyte ratio (NLR) is a simple marker of inflammation obtained from routine complete blood counts that predicts prognosis in a variety of cardiovascular diseases.^9^, ^10^, ^11^, ^12^ Elevated NLR or individual components are associated with worse outcome in patients with acute decompensated HF and HF patients awaiting transplant^13^, ^14^, ^15^, ^16^; however, its prognostic value has not been adequately characterized in a well‐defined cohort of HFrEF and HFpEF patients with new‐onset or worsening symptoms. Furthermore, no study has elucidated the relationship of NLR to other established inflammatory biomarkers in HF patients. As a marker representative of several pathways of systemic inflammation, the concept of NLR acting as a single, routinely available inflammatory biomarker is particularly attractive.

The aims of this study were to evaluate the association of NLR with biomarkers related to various inflammatory pathways in patients with HF, to determine its association with outcomes in both HFrEF and HFpEF in two independent cohorts and to assess its incremental value in addition to clinical variables.

## Methods

### Primary cohort

The primary study cohort included participants from the validation cohort of the Systems Biology Study to Tailored Treatment in Chronic Heart Failure (BIOSTAT‐CHF). The full details of BIOSTAT‐CHF validation cohort including inclusion/exclusion criteria have been published previously.^17^ In brief, BIOSTAT‐CHF was a multicentre, prospective, observational study that included both an index cohort of 2516 chronic HF patients with worsening signs and/or symptoms from 11 European countries, who were considered to be on suboptimal medical treatment, and a validation cohort of 1738 HF patients was recruited from six centres in Scotland. The recruitment period started in October 2010 and was completed in April 2014. NLR was only measured in the validation cohort of BIOSTAT‐CHF, and so, this cohort acted as the primary cohort for this study. Inclusion criteria for the BIOSTAT‐CHF validation cohort were patients ≥18 years old with diagnosed HF with either echocardiographic evidence of left ventricular systolic dysfunction or a previous documented admission to hospital with HF requiring furosemide, currently treated with furosemide ≥20 mg/day or equivalent, and not previously treated or receiving ≤50% of target doses of ACEI/ARB and/or beta‐blocker according to the 2008 ESC guidelines.^18^ Patients with acute myocarditis were excluded. Patients were recruited from both the inpatient and outpatient setting. BIOSTAT‐CHF complied with the Declaration of Helsinki, medical ethics committees of participating centres approved the study, and all participants provided written informed consent prior to participation.

Available medical history, demographics, current medications, lab measurements including full blood counts (FBC) and a panel of biomarkers were obtained at the baseline visit. Ninety‐two biomarkers related to cardiovascular processes were evaluated using the Olink Cardiovascular and Oncology panels as previously described.^19^ Measurement of these biomarkers was performed by Olink Bioscience analysis service using the Proseek Multiplex Inflammatory 96 × 96 kit, with additional real‐time PCR for further quantification. For this study, we focussed on biomarkers primarily related to inflammation, summarized in *Table* *S1*.^20^ NLR was calculated from baseline FBC. There were no patients with known active haematological malignancy recorded; however, as data on malignancy (in particularly haematological malignancy) were not routinely obtained,^21^ we excluded patients with a lymphocyte count > 8 × 10^9^/L on the assumption that this might be suggestive of an undiagnosed haematological condition. Echocardiographic data were used to stratify patients into reduced (LVEF < 40%) and preserved (LVEF ≥ 50%) ejection fraction.

### Validation cohort

The validation cohort for this study included participants from the Genetics of Diabetes and Audit Research Tayside Scotland (GoDARTS). The full details of the GoDARTS study have been published previously.^22^ Briefly, GoDARTS is a database consisting of 18 306 participants (10 149 with Type 2 diabetes and 8157 healthy controls) from Tayside (Scotland, UK). The presence or absence of HF at baseline was not a criteria for inclusion or exclusion. Baseline data were collected on all patients including prescription data, echocardiography reports, demographic data and clinical variables. Patient data are linked back as far as 1987 through Community Health Index number. Collection and analysis of GoDARTS data was approved by the East of Scotland Research Ethics committee, in compliance with the Declaration of Helsinki.

Patients who survived to 30 days following their first recorded HF hospitalization (ICD‐10 code I50) were included in this study. NLR was calculated from FBC taken nearest the time of hospitalization. Baseline clinical, demographic and prescription data were obtained from electronic health records. Patients underwent echocardiography for clinical indications, and ejection fraction where available was used to stratify patients into those with reduced and preserved LVEF.^23^ LVEF was not always measured using Simpson's method in these clinical reports, so conversion of qualitative assessment of LVEF was made using the 2011 ACCF/AHA criteria,^24^ where moderate or severe left ventricular systolic dysfunction was estimated to be LVEF < 40%.

### Clinical outcomes

The primary outcome was all‐cause mortality and/or HF hospitalization. In BIOSTAT‐CHF, all deaths and hospitalizations were recorded by the local investigator,^25^ with death and HF hospitalization outcome data also supplemented by electronic health records using ICD‐10 coding (I50) from the Scottish Morbidity Record (SMR) and General Register Office Scotland (GRO). In GoDARTS, all clinical outcomes were obtained from electronic health record data using the SMR and GRO. Time to death or subsequent HF hospitalization following the index HF hospitalization was ascertained. In both cases, outcomes were not centrally adjudicated.

### Statistical analysis

Baseline variables and clinical outcomes were recorded and categorized by the median NLR. Continuous variables were reported as mean ± standard deviation (SD) or median and interquartile range and categorical data as number and percentage. Correlations between NLR and biomarkers were determined using the Spearman correlation. The association between NLR and the primary outcome was determined using survival analysis (Cox proportional hazard model and Kaplan–Meier analysis) adjusting for the BIOSTAT‐CHF risk prediction model, which includes age, HF hospitalization in the previous year, peripheral oedema, systolic blood pressure, estimated glomerular filtration rate, urea, *N*‐terminal pro‐brain natriuretic peptide, haemoglobin, high‐density lipoprotein cholesterol, sodium and beta‐blocker use.^26^ This model performed similarly in both HFrEF and HFpEF with C‐statistics for prediction of the primary outcome of 0.68 in HFrEF and 0.69 in HFpEF. The cohort was further stratified into tertiles of NLR to determine the stepwise association with increasing NLR and the primary outcome. We conducted interaction tests to see if the association between NLR and outcomes differed based on ejection fraction and place of recruitment (i.e. patients recruited from the inpatient vs. outpatient setting). As ejection fraction was not available in the whole BIOSTAT cohort, we performed sensitivity analyses excluding those without available ejection fraction. In BIOSTAT, we have also found several other biomarkers, specifically cancer antigen 125 (CA‐125),^27^ fibroblast growth factor 23 (FGF‐23),^28^ bioadrenomedullin^29^ and interleukin 6,^30^ that have been associated with adverse outcomes; therefore, we also conducted an analysis to determine the association of NLR with outcomes after adjustment for these biomarkers. As NLR has previously been shown to be associated with markers of congestion, we also performed a multivariable analysis with adjustment for pulmonary congestion, hepatomegaly and elevated jugular venous pressure in addition to the BIOSTAT‐CHF risk model. Finally, NLR was added to BIOSTAT‐CHF risk score, and improvements in predictive capability were assessed using C‐statistic, integrated discrimination index (IDI) and net reclassification index (NRI).

A Cox proportional hazard model and Kaplan–Meier analysis were used to determine the association between NLR and the primary outcome in the validation cohort. As not all variables required for the BIOSTAT‐CHF risk model were available, a pragmatic multivariable adjustment for age, gender, diabetes, cholesterol, blood pressure, smoking status, ACEI/ARB and beta‐blocker use at the time of diagnosis and prior myocardial infarction was used. Statistical analyses were performed using SPSS Version 22.0 and R Version 3.5.1. A *P*‐value < 0.05 was considered significant.

## Results

### Primary cohort (BIOSTAT‐CHF): Baseline characteristics

In total, 1625 patients had NLR measured in the BIOSTAT cohort. Of these, three patients had a lymphocyte count above 8 × 10^9^/L and thus were excluded from analysis in case this represented an undiagnosed haematological condition. Baseline characteristics of the 1622 patients analysed are summarized in *Table* *1*. The mean age of the cohort was 74 ± 10 years, and the majority of patients were male (67%). Patients were predominantly NYHA Classes 2–3 (86%) and had ischaemic HF aetiology (65%). There were 890 patients with LVEF < 40% (HFrEF) and 526 with LVEF ≥ 50% (HFpEF). As per study inclusion criteria, almost all patients were receiving loop diuretics, whereas 70.8% were receiving an ACEI/ARB and 73.2% were on beta‐blocker therapy. The median NLR was 3.22. Patients with NLR above the median value were older and more likely to be male. Despite having a shorter duration of HF, they also had higher NT‐proBNP and were more symptomatic with higher NYHA class and more clinical congestion. They were also less likely to be taking ACEI/ARB or beta‐blockers than patients with NLR below the median (ACEI/ARB: 65.6% vs. 75.9%, *P* < 0.001; beta‐blockers: 69.7% vs. 76.8%, *P* = 0.002). There were no significant differences in most baseline comorbidities, although they were more likely to have renal impairment. Eight hundred forty‐seven patients were recruited from the inpatient setting, whereas 778 were outpatients with worsening HF; NLR was significantly higher in inpatients than outpatients (4.69 vs. 3.33, *P* < 0.001).

**Table 1:** Baseline characteristics of the BIOSTAT‐CHF cohort by stratified by median NLR

	All patients(*n* = 1,622)	NLR < 3.22 (*n* = 807)	NLR ≥ 3.22 (*n* = 815)	*P*‐value
Demographics				
Age (years)	74 ± 10	72 ± 11	75 ± 11	**<0.001**
Female	536 (33.0)	300 (37.2)	236 (29.0)	**<0.001**
Current smoker	562 (34.6)	269 (33.3)	293 (36.0)	0.14
BMI (kg/m^2^)	29.1 ± 6.4	29.7 ± 6.3	28.6 ± 6.4	**0.001**
Clinical profile				
NYHA class, %				**<0.001**
I	16 (1.0)	6 (0.7)	10 (1.2)	
II	680 (41.9)	413 (51.2)	267 (32.8)	
III	713 (44.0)	326 (40.4)	387 (47.5)	
IV	212 (13.1)	61 (7.6)	151 (18.5)	
Duration of HF (years)	5.3 ± 4.7	5.6 ± 4.7	5.0 ± 4.7	**0.017**
Ischaemic aetiology of HF	1,056 (65.1)	530 (65.7)	526 (64.5)	0.64
Systolic blood pressure, (mmHg)	125 ± 21	126 ± 22	125 ± 23	0.50
Diastolic blood pressure (mmHg)	69 ± 12	70 ± 12	68 ± 14	**0.003**
Heart rate (bpm)	76 ± 22	74 ± 21	78 ± 22	**<0.001**
Inpatient	847 (52.2)	340 (42.1)	507 (62.2)	**<0.001**
Elevated jugular venous pressure	422 (26.0)	162 (20.1)	260 (31.9)	**<0.001**
Pulmonary congestion	661 (40.8)	252 (31.2)	409 (50.2)	**<0.001**
Hepatomegaly	58 (3.6)	20 (2.5)	38 (4.7)	**0.032**
Peripheral oedema	885 (54.6)	374 (46.3)	511 (62.7)	**<0.001**
Echocardiographic measurements				
Left ventricular ejection fraction (%)	41 ± 14	40 ± 13	41 ± 14	0.76
LVEF < 40%	523 (32.2)	255 (31.6)	268 (32.9)	
LVEF ≥ 40%	662 (40.8)	315 (39.0)	347 (42.6)	
No LVEF available	437 (26.9)	237 (29.4)	200 (24.5)	
Past medical history				
Myocardial infarction	794 (49.0)	395 (48.9)	399 (49.0)	0.96
Atrial fibrillation	713 (44.0)	340 (42.1)	373 (45.8)	0.17
Diabetes mellitus	522 (32.2)	251 (31.1)	271 (33.3)	0.34
Hypertension	937 (57.8)	449 (55.6)	488 (59.9)	0.09
Device therapy				0.73
Pacemaker	109 (6.7)	55 (6.8)	54 (6.6)	
ICD	65 (4.0)	28 (3.5)	37 (4.5)	
CRT	79 (4.9)	37 (4.6)	42 (5.2)	
Medication				
ACEI/ARB	1,148 (70.8)	613 (76.0)	535 (65.6)	**<0.001**
Beta‐blockers	1,188 (73.2)	620 (76.8)	568 (69.7)	**0.001**
Aldosterone antagonists	528 (32.6)	252 (31.2)	276 (33.9)	0.28
Loop diuretics	1,574 (97.0)	783 (97.0)	791 (97.1)	>0.99
Laboratory measurements				
Creatinine (μmol/L)	111 ± 45	102 ± 42	118 ± 55	**<0.001**
Urea (mmol/L)	8.6 (6.0–11.3)	8.4 (5.9–11.0)	8.8 (6.0–11.7)	0.12
Estimated glomerular filtration rate (ml/min/1.73 m^2^)^a^				**<0.001**
≥60	855 (52.7)	481 (59.6)	374 (45.9)	
45–59	371 (22.9)	178 (22.1)	193 (23.7)	
<45	393 (24.2)	145 (18.0)	248 (30.4)	
NT‐proBNP (ng/L)	1243 (0–2670)	834 (0–1684)	2065 (0–4319)	**<0.001**
Neutrophils (10^9^/L)	5.4 ± 2.6	4.4 ± 1.4	6.5 ± 3.0	**<0.001**
Lymphocytes (10^9^/L)	1.6 ± 0.9	2.0 ± 1.1	1.2 ± 0.5	**<0.001**
Haemoglobin (g/d)	15.1 ± 2.7	15.8 ± 2.5	14.5 ± 2.8	0.08
Platelets (10^9^/L)	245 ± 92	236 ± 81	253 ± 101	**<0.001**

ACEI, angiotensin‐converting enzyme inhibitor; ARB, angiotensin II receptor blocker; CRT, cardiac resynchronization therapy; ICD, implantable cardioverter defibrillator.

Bold indicates *P* < 0.05. Continuous variables reported as mean ± standard deviation or median (interquartile range), and categorical variables as number (percentage).

^a^ Three patients in NLR < 3.22 group did not have eGFR measured.

### Correlations between NLR and biomarkers related to inflammation

**Figure 1:**
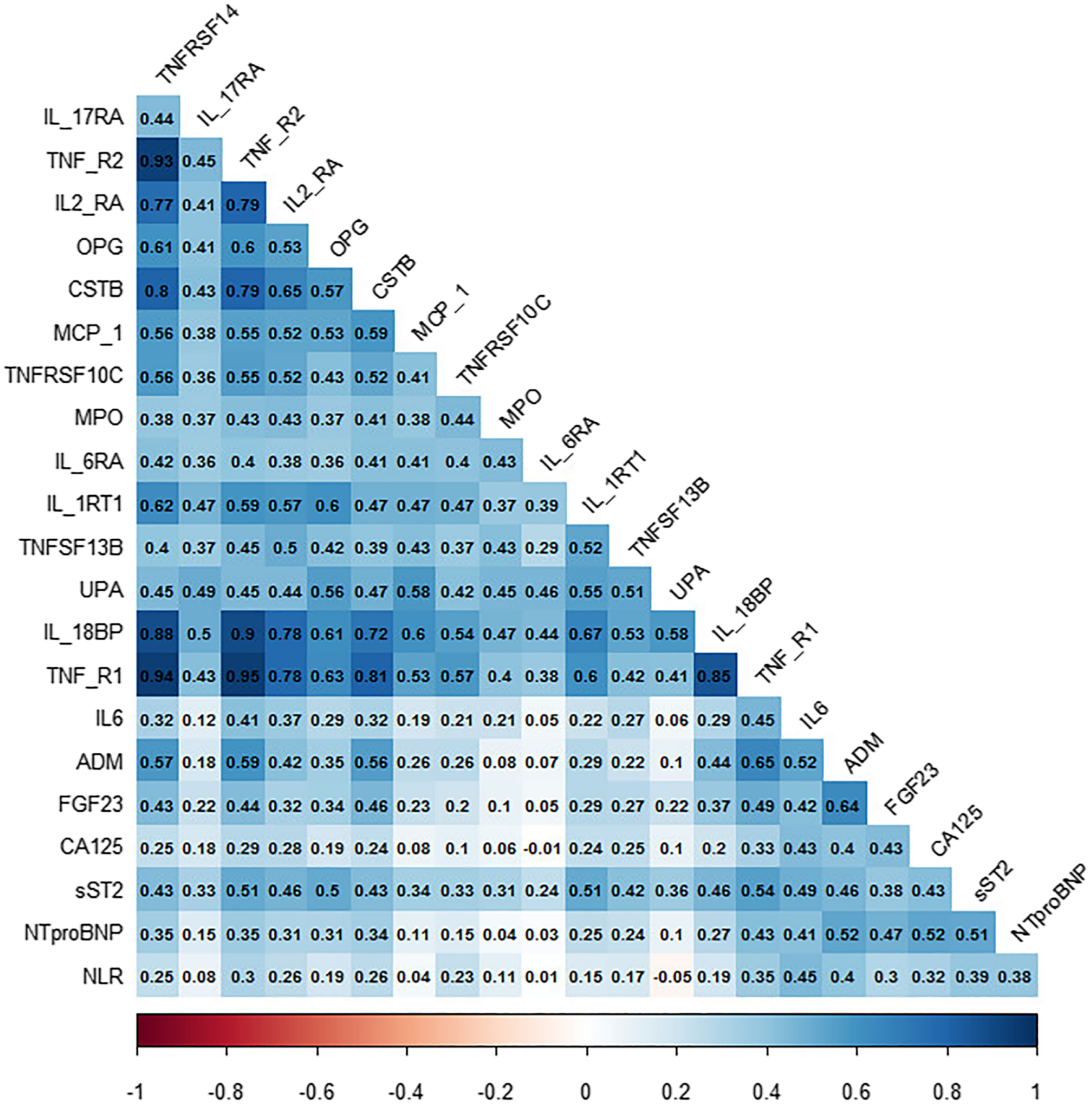
Correlation matrix of NLR, NT‐proBNP and biomarkers of inflammation. Correlation matrix showing Spearman correlation of biomarkers.

Correlations between NLR and biomarkers are summarized in *Figure* *1*. NLR was most strongly associated with interleukin 6 (ρ = 0.41), bio‐adrenomedullin (ρ = 0.40), sST2 (ρ = 0.39), NT‐proBNP (ρ = 0.38), tumour necrosis factor (TNF) receptor 1(ρ = 0.35), CA‐125 (ρ = 0.32) and TNF receptor 2 (ρ = 0.30), all *P* < 0.001. Biomarker correlations were similar stratified by LVEF **(** *Figure* *S1* **)**.

### Association between NLR and clinical outcomes in BIOSTAT‐CHF

Associations between NLR and outcomes in the primary cohort are summarized in *Table* *2* **.** Over the median follow‐up period of 18 months, the primary outcome occurred in 667 individuals, including 447 deaths and 406 HF hospitalizations. After adjustment for the BIOSTAT‐CHF risk prediction model, NLR was significantly associated with increased incidence of the primary outcome (hazard ratio [HR] 1.18 per SD increase; 95% confidence interval [CI] 1.11–1.25, *P* < 0.001). Both elevated neutrophil count (HR 1.11 per SD increase; 95% CI 1.04–1.19, *P* = 0.002) and lower lymphocyte count (HR 0.85 per SD increase; 95% CI 0.76–0.95, P = 0.005) were also associated with the primary outcome. NLR was also significantly associated with increased all‐cause mortality (HR 1.37; 95% CI 1.30–1.44, *P* < 0.001).

**Table 2:** Association between neutrophil count, lymphocyte count, NLR and outcomes in BIOSTAT‐CHF

	Whole cohort (*n* = 1622)		LVEF < 40% (*n* = 523)		LVEF ≥ 40% (*n* = 662)	
	Hazard ratio (95% CI)	*P*‐value	Hazard ratio (95% CI)	*P*‐value	Hazard ratio (95% CI)	*P*‐value
Mortality and/or HF hospitalization						
Neutrophil count per SD increase	1.11 (1.04–1.20)	**0.001**	1.24 (1.12–1.37)	**<0.001**	1.02 (0.91–1.14)	0.72
Lymphocyte count per SD increase	0.74 (0.65–0.85)	**<0.001**	0.59 (0.46–0.75)	**<0.001**	0.81 (0.65–0.99)	**0.041**
NLR per SD increase	1.18 (1.11–1.26)	**<0.001**	1.24 (1.12–1.35)	**<0.001**	1.10 (0.99–1.23)	0.07
NLR Tertile 1	Baseline		Baseline		Baseline	
NLR Tertile 2	1.33 (1.06–1.67)	**0.014**	1.98 (1.30–3.01)	**0.001**	1.12 (0.87–1.46)	0.42
NLR Tertile 3	1.72 (1.37–2.15)	**<0.001**	2.75 (1.84–4.09)	**<0.001**	1.51 (1.05–2.16)	**0.026**
Mortality						
Neutrophil count per SD increase	1.21 (1.14–1.29)	**<0.001**	1.46 (1.31–1.63)	**<0.001**	1.03 (0.90–1.17)	0.69
Lymphocyte count per SD increase	0.40 (0.33–0.48)	**<0.001**	0.36 (0.26–0.49)	**<0.001**	0.58 (0.44–0.76)	**<0.001**
NLR per SD increase	1.38 (1.30–1.46)	**<0.001**	1.38 (1.26–1.51)	**<0.001**	1.18 (1.06–1.33)	**0.004**
NLR Tertile 1	Baseline		Baseline		Baseline	
NLR Tertile 2	2.14 (1.55–2.95)	**<0.001**	4.06 (2.16–7.60)	**<0.001**	1.51 (0.92–2.48)	0.10
NLR Tertile 3	4.14 (3.10–5.55)	**<0.001**	7.71 (4.32–13.76)	**<0.001**	2.30 (1.44–3.68)	**<0.001**

All hazard ratios adjusted for the BIOSTAT‐CHF risk model, which includes age, HF hospitalization in the previous year, peripheral oedema, systolic blood pressure, estimated glomerular filtration rate, urea, *N*‐terminal pro‐brain natriuretic peptide, haemoglobin, high‐density lipoprotein cholesterol, sodium and beta‐blocker use.

Bold indicates *P* < 0.05.

There was no significant interaction between NLR and LVEF category (<40% or ≥40%) for the association with the primary outcome (*P* = 0.06) or mortality (*P* = 0.27), with NLR significantly associated with the primary outcome in patients with LVEF < 40% (HR 1.24; 95% CI 1.12–1.35, *P* < 0.001), though the association did not quite reach statistical significance in patients with LVEF ≥40% (HR 1.10; 95% CI 0.99–1.23, *P* = 0.07). NLR was more strongly associated with the primary outcome in outpatients than those recruited as inpatients (HR 1.48; 95% CI 1.30–1.69, *P* < 0.001 vs. 1.11; 95% CI 1.03–1.19, *P* = 0.005, respectively; interaction *P‐*value < 0.001). In a model including the BIOSTAT risk score and NT‐proBNP as an additional variable, NLR remained associated with both the primary outcome (HR 1.17; 9% CI 1.10–1.24, *P* < 0.001) and mortality only (HR 1.32; 95% CI 1.25–1.40, *P* < 0.001).

**Figure 2:**
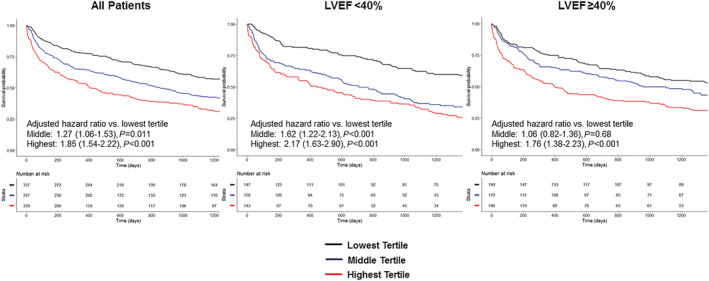
Outcomes stratified by tertiles of NLR in the BIOSTAT‐CHF cohort. Kaplan–Meier analysis of mortality/HF hospitalization stratified by NLR tertile in the whole BIOSTAT‐CHF cohort and in those with HFrEF and HFpEF.

When the cohort was stratified into tertiles of NLR, after adjustment for the BIOSTAT‐CHF risk score, there was a significant stepwise association between higher NLR and increased likelihood of the primary outcome (Tertile 2: HR 1.33; 95% CI 1.06–1.67, *P* = 0.014; Tertile 3: HR 1.72; 95% CI 1.37–2.15, *P* < 0.001) (*Figure* *2*), with similar results for mortality alone. Results followed a similar pattern when the cohort was stratified by LVEF, with patients in the highest tertile having worse outcome regardless of LVEF (LVEF < 40%: HR 2.75; 95% CI 1.84–4.09, *P* < 0.001; LVEF ≥ 40%: HR 1.51; 95% CI 1.05–2.16, *P* = 0.026). This pattern was similar in patients with unknown LVEF (*Table S2*).

NLR remained significantly associated with the primary outcome after additional adjustment for markers of congestion (HR 1.22; 95% CI 1.14–1.31, *P* < 0.001).

There was no significant interaction with HF treatment at baseline in patients with LVEF < 40% for the association between NLR and the primary outcome (ACEI/ARB *P* = 0.22; beta‐blocker *P* = 0.44; MRA *P* = 0.29).

When NLR was added to the BIOSTAT‐CHF risk score, there was a marginal increase in C‐statistic for prediction of the primary outcome from 0.716 to 0.72 but no difference in mortality (0.755–0.754). In contrast, the C‐statistic for prediction of the primary outcome when neutrophils or lymphocytes were individually added to the BIOSTAT‐CHF risk score was unchanged at 0.716. There were significant improvements in IDI and continuous NRI at 2 years with addition of NLR to the BIOSTAT risk model for both the primary outcome (IDI 0.009; 95% CI 0.00–0.019, *P* = 0.030; continuous NRI 0.112, 95% CI 0.012–0.176, *P* = 0.040) and for mortality alone (IDI 0.050; 95% CI 0.031–0.072, *P* < 0.001; continuous NRI 0.282, 95% CI 0.227–0.340, *P* < 0.001).

### Association of NLR and outcomes compared with other biomarkers

After adjustment for the BIOSTAT‐CHF risk score and biomarkers related to inflammation that were also associated with the primary outcome at *P* < 0.005 (cystatin B, interleukins 1, 6 and 17, osteoprotegerin, TNF receptors 1 and 2 and TNF receptor superfamily members 13B and 14), NLR remained significantly associated with the primary outcome (HR 1.17; 95% CI 1.09–1.25, *P* < 0.001) as summarized in *Table* *3*.

**Table 3:** Association of inflammatory biomarkers with the primary outcome adjusted for the BIOSTAT‐CHF model

Biomarker	Hazard ratio per SD increase (95% CI) (adjusted for BIOSTAT risk score only)	*P*‐value	Hazard ratio per SD increase (adjusted for BIOSTAT risk score + significant inflammatory biomarkers)	*P*‐value
NLR	1.18 (1.11–1.25)	**<0.001**	1.15 (1.07–1.24)	**<0.001**
CSTB	1.15 (1.06–1.24)	**<0.001**	1.10 (0.98–1.24)	0.12
IL6	1.16 (1.08–1.26)	**<0.001**	1.10 (1.00–1.21)	**0.039**
IL17RA	1.13 (1.04–1.22)	**0.002**	1.06 (0.96–1.18)	0.22
IL18BP	1.07 (0.98–1.16)	0.13		
IL1RT1	1.10 (1.02–1.20)	**0.019**	0.95 (0.83–1.08)	0.44
IL1RT2	1.06 (0.98–1.14)	0.17		
IL2RA	1.07 (0.99–1.17)	0.09		
IL6RA	0.98 (0.91–1.05)	0.56		
MCP1	1.04 (0.97–1.12)	0.24		
MPO	1.06 (0.99–1.14)	0.11		
OPG	1.12 (1.04–1.22)	**0.005**	1.00 (0.89–1.13)	0.99
TNFR1	1.17 (1.07–1.28)	**<0.001**	1.16 (0.84–1.61)	0.38
TNFR2	1.13 (1.03–1.23)	**0.006**	0.70 (0.54–0.92)	**0.009**
TNFRSF10C	1.03 (0.96–1.12)	0.40		
TNFRSF14	1.13 (1.04–1.22)	**0.005**	1.17 (0.88–1.54)	0.28
TNFSF13B	1.17 (1.09–1.27)	**<0.001**	1.17 (1.06–1.29)	**0.001**
UPA	1.04 (0.96–1.12)	0.37		

CSTB, cystatin‐B; IL17RA, interleukin 17 receptor A; IL18BP, interleukin 18 binding protein; IL1RT1, interleukin 1 receptor type 1; IL1RT2, interleukin 1 receptor type 2; IL2RA, interleukin 2 receptor subunit alpha; IL6, interleukin 6; IL6RA, interleukin 6 receptor subunit alpha; MCP1, monocyte chemotactic protein 1; MPO, myeloperoxidase; OPG, osteoprotegerin; NLR, neutrophil‐to‐lymphocyte ratio; TNFR1, tumour necrosis factor receptor 1; TNFR2, tumour necrosis factor receptor 2; TNFRSF14, tumour necrosis factor receptor superfamily member 14; TNFSF13B, tumour necrosis factor ligand superfamily member 13B; TNRSF10C, tumour necrosis factor receptor superfamily member 10C; UPA, urokinase plasminogen activator.

Adjusted for the BIOSTAT‐CHF risk model, which includes age, HF hospitalization in the previous year, peripheral oedema, systolic blood pressure, estimated glomerular filtration rate, urea, *N*‐terminal pro‐brain natriuretic peptide, haemoglobin, high‐density lipoprotein cholesterol, sodium, and beta‐blocker use.

Bold indicates *P* < 0.05.

In a separate model adjusted for the BIOSTAT‐CHF risk score, NT‐proBNP, CA‐125, FGF‐23, bio‐adrenomedullin and interleukin 6, NLR remained significantly associated with both the primary outcome (HR 1.13; 95% CI 1.04–1.20, *P* = 0.002) and mortality alone (HR 1.14; 95% CI 1.07–1.23, *P* < 0.001) (*Table S3*).

### Validation cohort (GoDARTS): Baseline characteristics

In total, 1013 patients were available for analysis from the GoDARTS cohort. Again, three were excluded as they had a lymphocyte count > 8 × 10^9^/L. Baseline characteristics are summarized in *Table S4*. The mean age of the cohort was 74 ± 10, and the majority of patients were male (686, 67.7%). There were 448 patients with LVEF < 40% and 565 with LVEF ≥ 40%. The majority of patients were receiving HF medications including ACE/ARB (59.9%) and beta‐blocker therapy (58.8%). The median NLR was 4.29. The majority of patients (964, 94.8%) had their NLR measured within 7 days of their index hospitalization (median time 0 days, interquartile range 0–0).

### Association between NLR and clinical outcomes in GoDARTS

Associations between NLR and outcomes in GODARTS patients are summarized in *Table* *4* **.** Over the median follow‐up period of 22 months, the primary outcome occurred in 759 individuals, including 641 deaths and 409 HF hospitalizations. After multivariable adjustment, NLR was significantly associated with increased incidence of the primary outcome (HR 1.20 per SD increase; 95% CI 1.13–1.27, *P* < 0.001). NLR was also significantly associated with increased all‐cause mortality (HR 1.20 per SD increase; 95% CI 1.13–1.27, *P* < 0.001). In line with the BIOSTAT‐CHF cohort, NLR was significantly associated with the primary outcome regardless of LVEF (LVEF < 40%: HR 1.20 per SD increase; 95% CI 1.11–1.30, *P* < 0.001) and HFpEF patients (HR 1.27 per SD increase; 95% CI 1.15–1.39, *P* < 0.001).

**Table 4:** Association between tertiles of NLR and outcomes stratified in GoDARTS

	Whole cohort (*n* = 1013)		LVEF < 40% (*n* = 448)		LVEF ≥ 40% (*n* = 565)	
	Hazard ratio (95% CI)	*P*‐value	Hazard ratio (95% CI)	*P*‐value	Hazard ratio (95% CI)	*P*‐value
Mortality and/or HF hospitalization						
NLR per SD increase	1.20 (1.13–1.27)	**<0.001**	1.20 (1.11–1.30)	**<0.001**	1.27 (1.15–1.39)	**<0.001**
Tertile 1	Baseline		Baseline		Baseline	
Tertile 2	1.27 (1.06–1.53)	**0.011**	1.62 (1.22–2.13)	**<0.001**	1.06 (0.82–1.36)	0.68
Tertile 3	1.85 (1.54–2.22)	**<0.001**	2.17 (1.63–2.90)	**<0.001**	1.76 (1.38–2.23)	**<0.001**
Mortality						
NLR per SD increase	1.20 (1.13–1.27)	**<0.001**	1.17 (1.07–1.28)	**<0.001**	1.29 (1.17–1.42)	**<0.001**
Tertile 1	Baseline		Baseline		Baseline	
Tertile 2	1.21 (0.99–1.49)	**0.07**	1.41 (1.03–1.94)	**0.034**	1.07 (0.82–1.40)	0.62
Tertile 3	1.86 (1.53–2.26)	**<0.001**	2.12 (1.54–2.91)	**<0.001**	1.71 (1.33–2.21)	**<0.001**

Adjusted for age, gender, diabetes, cholesterol, blood pressure, smoking status, ACEI/ARB and beta‐blocker use at the time of diagnosis, and prior myocardial infarction

Bold indicates *P* < 0.05.

When the cohort was stratified into tertiles of NLR, after multivariable adjustment for age, gender, diabetes, cholesterol, blood pressure, smoking status, ACEI/ARB and beta‐blocker use and prior myocardial infarction, there was a significant stepwise association between higher NLR and increased likelihood of the primary outcome (Tertile 2: HR 1.27; 95% CI 1.06–1.53, *P* = 0.011; Tertile 3: HR 1.85; 95% CI 1.54–2.22, *P* < 0.001), with similar results for mortality alone (*Figure S2*). These results were similar in patients with LVEF ≥ 40% and those with LVEF < 40%.

**Figure 3:**
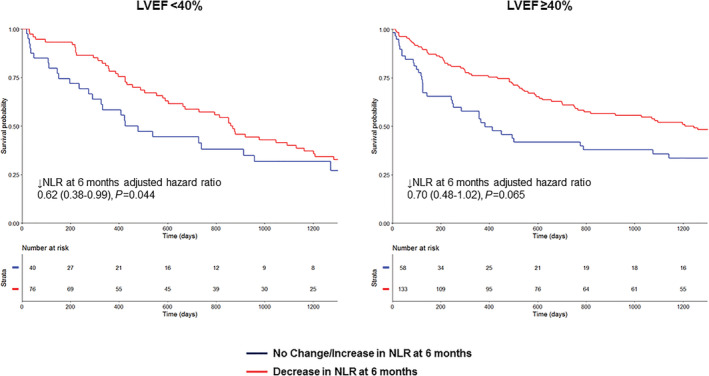
Outcomes in the GoDARTS cohort stratified by change in NLR at 6 months in HFrEF and HFpEF. Kaplan–Meier analysis of mortality/HF hospitalization stratified by NLR change at 6 months in GoDARTS.

Three hundred seven individuals had not died or had a HF hospitalization at 6 months and had an available NLR at this time point (116 LVEF < 40%, 191 LVEF ≥ 40%; 175 male). Median NLR in this subset of patients was 3.23 (interquartile range 0.62–5.84). NLR had decreased compared with baseline in 209 patients (68.1%). After adjustment, a decrease in NLR at 6 months was significantly associated with reduced incidence of mortality and/or HF hospitalization (HR 0.75; 95% CI 0.57–0.98, *P* = 0.036). The association between decrease in NLR at 6 months and improved outcome was similar regardless of LVEF (LVEF < 40%: HR 0.62; 95% CI 0.38–0.99, *P* = 0.044; LVEF ≥ 40%: HR 0.70; 95% CI 0.48–1.02, *P* = 0.065, interaction *P*‐value 0.60) (*Figure* *3*).

## Discussion

In this study, we identified several findings. Most importantly, we found that increased NLR was significantly associated with clinical outcomes in patients with new‐onset or worsening HFrEF and HFpEF and validated this in a second independent cohort. We also found that NLR was significantly correlated with several established biomarkers related to inflammatory pathways in HF. In addition, NLR was also significantly correlated with NT‐proBNP. Finally, we have shown that a decrease in NLR at 6 months was associated with reduced incidence of mortality/HF hospitalization.

Our findings add to the accumulating evidence base showing the value of NLR as a prognostic marker in cardiovascular disease.^9^, ^10^, ^11^ In HF patients specifically, previous studies have demonstrated the relationship between NLR and outcome in patients with acute decompensated HF and HF patients awaiting transplant.^13^, ^14^, ^31^, ^32^ Boralkar et al.^33^ investigated 443 patients hospitalized with acute HF and preserved ejection fraction and found that NLR was associated with all‐cause mortality. Yan et al.^34^ investigated 1335 elderly patients with chronic HF and found that raised NLR was associated with major cardiac events including cardiac death and rehospitalization. Our multicentre study composed of two large independent cohorts extends these findings by demonstrating the association of NLR with adverse outcomes in both HFrEF and HFpEF patients in both an inpatient and outpatient setting over a long follow‐up period. We have also shown that NLR is higher in inpatients than outpatients, suggesting that inflammation may play a part in HF decompensations. Supporting this, we found that elevated NLR was also associated with higher NT‐proBNP and markers of congestion and worse NYHA functional class, despite similar LVEF and regardless of inpatient vs. outpatient setting.

We demonstrated a strong association between NLR and various biomarkers related to inflammatory pathways in HF that have been shown to independently predict prognosis in HF.^4^, ^5^, ^6^, ^7^, ^30^ These associations have been previously identified in other conditions such as end‐stage renal disease,^35^ and we have shown for the first time that this correlation is consistent in HF. The concept of NLR acting as a routinely available parameter of inflammatory status, correlating with other established inflammatory biomarkers and being independently associated with outcome with evidence of incremental prognostic value in addition to NT‐proBNP, is particularly appealing for clinical translation. NLR reflects both neutrophilia, typically representative of non‐specific inflammation, and lymphopaenia, a marker of physiological stress thought to be caused by programmed cell death as a result of oxidative stress and elevated plasma cortisol levels.^9^ There are two main pathways by which elevated NLR could be associated with worse outcome in HF: First, increased release of pro‐inflammatory cytokines by neutrophils could impair myocardial function by direct damage.^36^ Second, elevated NLR might also be reflective of increased sympathetic tone, one of the key pathophysiological mechanisms underlying HF.^37^ We also showed that NLR seems to have additional value compared with just neutrophil or lymphocyte count alone. NLR was associated with outcome independent of markers of congestion that we have previously reported in BIOSTAT‐CHF but was more strongly associated with prognosis than interleukin 6 (and any other specific inflammatory markers). Inflammation in HF is likely to be the result of several pathways, and so, a biomarker such as NLR that can identify these may be more useful than one at the end of a specific pathophysiological pathway. Indeed, supporting this, we found that NLR did provide improve discrimination to the BIOSTAT‐CHF risk model, whereas interleukin 6 did not.^30^ It is also notable there was no significant interaction between the prognostic association of NLR and LVEF, though elevated NLR was more strongly associated with the primary outcome in HFrEF. Inflammation is strongly hypothesized to play an important role in HFpEF, but our findings suggest that it may also be important in HFrEF, and further studies should be performed to elucidate this more clearly and in particular whether elevated NLR and inflammation is truly an underlying driver of HF or simply a marker of advanced disease.^2^, ^3^, ^38^, ^39^

Our finding of improved outcome in patients with decreased NLR at 6 months post‐HF hospitalization is particularly interesting as it may suggest a possibility for targeting inflammation, particularly in HFpEF, where there are no evidence‐based therapies as yet. Previous trials involving anti‐inflammatory targeting in HF have been largely unsuccessful, although many of these trials have been in HFrEF patients without specifically targeting those with evidence of high levels of systemic inflammation.^40^, ^41^, ^42^, ^43^ In RENEWAL, the soluble TNF antagonist etanercept was found to have no clinical benefit on death of HF hospitalization in HFrEF patients.^40^ This study reported soon after the ATTACH trial, in which a similar approach using infliximab in HFrEF patients also did not show any benefit and in fact potentially had deleterious effects at higher doses.^44^ Conversely, in CANTOS, increasing doses of canakinumab (an IL‐1β inhibitor) were associated with fewer HF hospitalizations.^45^ Patients in the canakinumab arm who achieved lower levels of high‐sensitivity C‐reactive protein (hs‐CRP) had improved outcome compared with those who did not achieve lower levels or those assigned to placebo, although only 20% of patients in CANTOS had a history of HF at baseline. NLR may have an advantage over other markers such as hs‐CRP as it is more readily available. In particular, use of a marker such as NLR might be used for patient selection in future trials of anti‐inflammatory therapies by identifying individuals with high levels of inflammation more likely to benefit from therapeutic anti‐inflammatory intervention.

Our study had a number of limitations. First, due to the observational study design and its inherent limitations, our findings serve to highlight the association between NLR and adverse outcome but cannot infer causation, although our study is strengthened by the use of two separate cohorts including both HFrEF and HFpEF. We do recognize that there were some differences between the cohorts, such as the fact that the GoDARTS cohort were all hospitalized patients whereas BIOSTAT‐CHF also included outpatients, and differences in ejection fraction measurements. NLR was recorded at baseline but not systematically during follow‐up, and so, we cannot exclude indication bias. Also, neutrophils do have rapid turnover and may be influenced by acute clinical events, so serial measurements at baseline might have been useful, although NLR was actually more strongly associated with outcome in outpatients in the BIOSTAT cohort. We did not systematically assess for the presence of undiagnosed malignancy in either cohort (e.g. with imaging), although there was no evidence of active malignancy in the electronic medical records of the patients. Nevertheless, we cannot completely exclude undiagnosed conditions that might affect NLR such as malignancy or rheumatological conditions. We did not measure other inflammatory markers such as C‐reactive protein or procalcitonin and did not perform other tests such as chest x‐rays to definitively exclude intercurrent infection. Nevertheless, all patients in the BIOSTAT‐CHF cohort had a clinical examination performed at baseline, and the consistency of our results across two cohorts and in both inpatients and outpatients does give further confidence that our results were not just driven by the presence of concomitant infection in all patients. Related to this, although patients with acute myocarditis were excluded, we did not systematically assess for the presence of chronic myocarditis, for example, with cardiovascular magnetic resonance imaging or endomyocardial biopsy. There may also be selection bias, and these results are not necessarily applicable to all HF patients, for example, hospitalized patients requiring haemodynamic support. Finally, as the GoDARTS cohort data and some BIOSTAT outcome data were obtained from electronic health records, we were not able to conduct a similar multivariable analysis using the BIOSTAT‐CHF risk prediction model. Theoretically, central adjudication of events may have provided more assurance around the documentation of HF hospitalizations, although use of electronic health records is well established and provides good accuracy.^23^, ^46^

## Conclusion

In HFrEF and HFpEF patients, NLR was strongly associated with biomarkers of inflammation and elevated levels were significantly associated with adverse outcome independent of other clinical variables. A decrease in NLR at 6 months was significantly associated with improved outcome in HFpEF patients. Future prospective randomized interventional studies are warranted to determine whether NLR can be used to select HF patients at high risk who might benefit from anti‐inflammatory therapies or might even be a treatment target in HF.

## Conflict of interest

We report no specific conflict of interest related to this study. S.D.A. reports receiving fees from Bayer, Boehringer Ingelheim, Cardiac Dimension, Impulse Dynamics, Novartis, Servier, St. Jude Medical and Vifor Pharma and grant support from Abbott Vascular and Vifor Pharma. A.A.V. reports personal fees from Amgen, personal fees from cytokinetics, personal fees from Boehringer Ingelheim, personal fees from Vifor, grants and personal fees from Roche, personal fees from Novartis, personal fees from Servier, personal fees from AstraZeneca, personal fees from Bayer, personal fees from GSK, personal fees from Myokardia and personal fees from Merck, outside the submitted work. L.L.N. and D.J.V. report grants from EU FP7 Program during the conduct of the study. C.C.L. received fees and/or research grants from Novartis, AstraZeneca and MSD. M.M. received consulting or speaker fees from Amgen, AstraZeneca, Bayer, Novartis, Relypsa, Servier, Stealth Therapeutics, Trevena and Abbott Vascular. GF reports grants from EU; Lecture and /or Committee member fees in trials sponsored by Bayer, Boehringer Ingelheim, Amgen, Medtronic, Novartis, Servier outside this work. All other authors have nothing to declare.

## Funding

This work was supported by a grant from the European Commission (FP7‐242209‐BIOSTAT‐CHF). The Wellcome Trust United Kingdom Type 2 Diabetes Case–Control Collection (GoDARTS [Genetics of Diabetes Audit and Research in Tayside]) was funded by the Wellcome Trust (072960/Z/03/Z, 084726/Z/08/Z, 084727/Z/08/Z, 085475/Z/08/Z and 085475/B/08/Z) and as part of the EU IMI‐SUMMIT program. IRM is supported by an NHS Education for Scotland/Chief Scientist Office Postdoctoral Clinical Lectureship (PCL 17/07).

## Supporting information


**Figure S1.** Correlation Matrix of NLR, NT‐proBNP and Biomarkers of Inflammation stratified by LVEF.
**Figure S2.** Outcomes Stratified by Tertiles of NLR in the GoDARTS Cohort.
**Table S1.** Summary of biomarkers reported in this study.
**Table S2.** Association of NLR with outcomes in patients with unknown LVEF in the BIOSTAT‐CHF cohort.
**Table S3.** Association of NLR and biomarkers previously reported to be associated with outcome in BIOSTAT‐CHF with outcomes in the BIOSTAT cohort adjusted for the BIOSTAT risk score and NT‐proBNP.
**Table S4.** Baseline characteristics of validation cohort (GoDARTS) by stratified by median NLR.
