# Correction to: ‘Neutrophil‐to‐lymphocyte ratio and outcomes in patients with new‐onset or worsening heart failure with reduced and preserved ejection fraction’

**DOI:** 10.1002/ehf2.14362

**Published:** 2023-03-31

In the paper by Curran *et al*.,^1^ the incorrect *Figure* *2* and *Figure* *S1* were published. There is no change to the data or text, and there is therefore no change to the message or interpretation of the manuscript.

**Figure 2:**
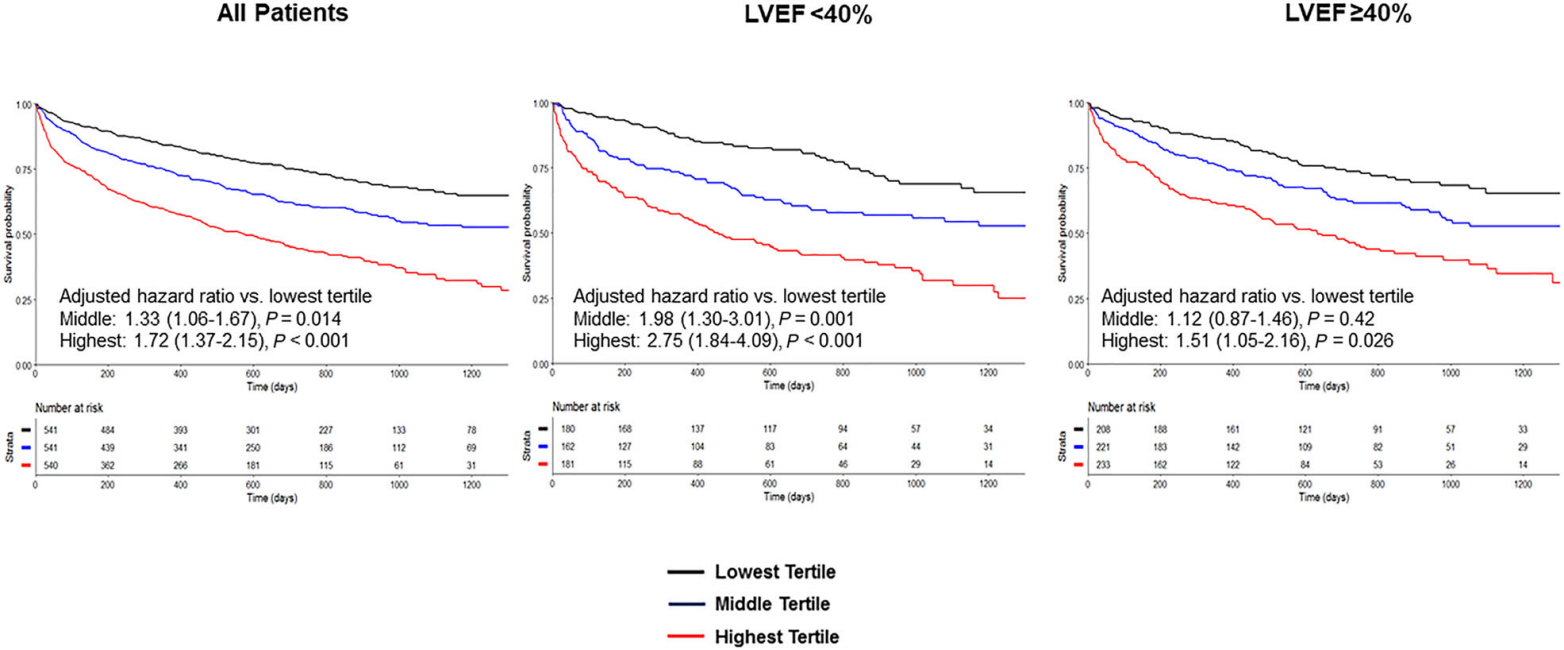
Outcomes stratified by tertiles of NLR in the BIOSTAT‐CHF cohort. Kaplan–Meier analysis of mortality/HF hospitalization stratified by NLR tertile in the whole BIOSTAT‐CHF cohort and in those with HFrEF and HFpEF.

The correct *Figure* *2* image should be:

The correct supporting information may be found online in the Supporting Information at the end of the original article.

The online version of the article has been corrected.

## Supporting information


**Figure S1.** Supporting Information.
