# Supplementary material for: Neutrophil‐to‐lymphocyte ratio and outcomes in patients with new‐onset or worsening heart failure with reduced and preserved ejection fraction
Source: ESC Heart Fail. 2021 May 16;8(4):3168–79. doi: 10.1002/ehf2.13424 (PMC8318449; doi:10.1002/ehf2.13424)
Supplement: Supplementary file 1 — Figure S1. Correlation Matrix of NLR, NT‐proBNP and Biomarkers of Inflammation stratified by LVEF. Figure S2. Outcomes Stratified by Tertiles of NLR in the GoDARTS Cohort. Table S1. Summary of biomarkers reported in this study. Table S2. Association of NLR with outcomes in patients with unknown LVEF in the BIOSTAT‐CHF cohort. Table S3. Association of NLR and biomarkers previously reported to be associated with outcome in BIOSTAT‐CHF with outcomes in the BIOSTAT cohort adjusted for the BIOSTAT risk score and NT‐proBNP. Table S4. Baseline characteristics of validation cohort (GoDARTS) by stratified by median NLR. [file EHF2-8-3168-s001.docx]

**Supplementary Figure 1.** Correlation Matrix of NLR, NT-proBNP and Biomarkers of Inflammation stratified by LVEF.

Correlation matrix showing Spearman correlation of biomarkers.


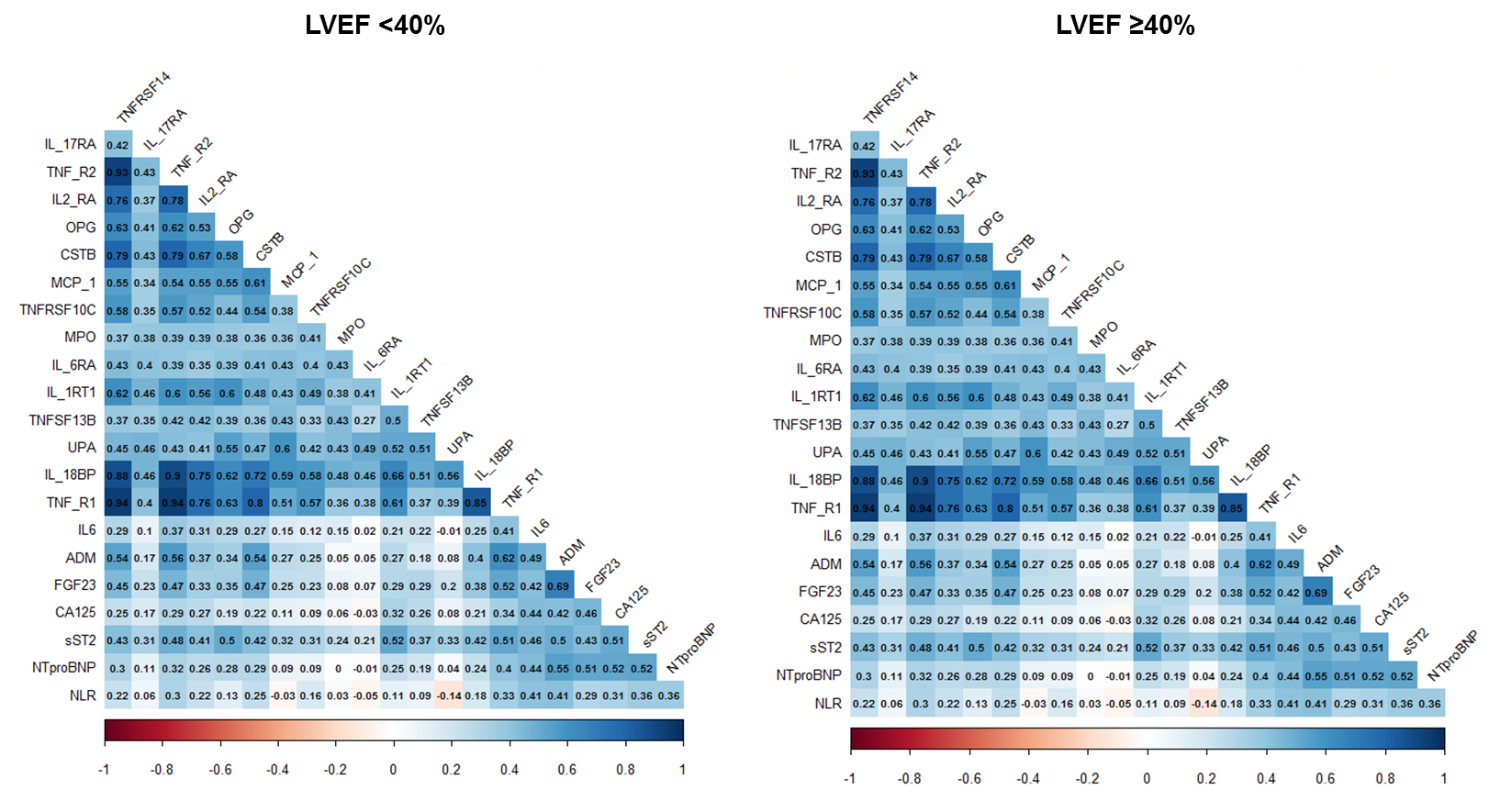


**Supplementary Figure 2.** Outcomes Stratified by Tertiles of NLR in the GoDARTS Cohort.

Kaplan-Meier Analysis of Mortality/HF Hospitalisation stratified by NLR tertile in the whole GoDARTS cohort and in those with LVEF <40% and LVEF ≥40%.

**
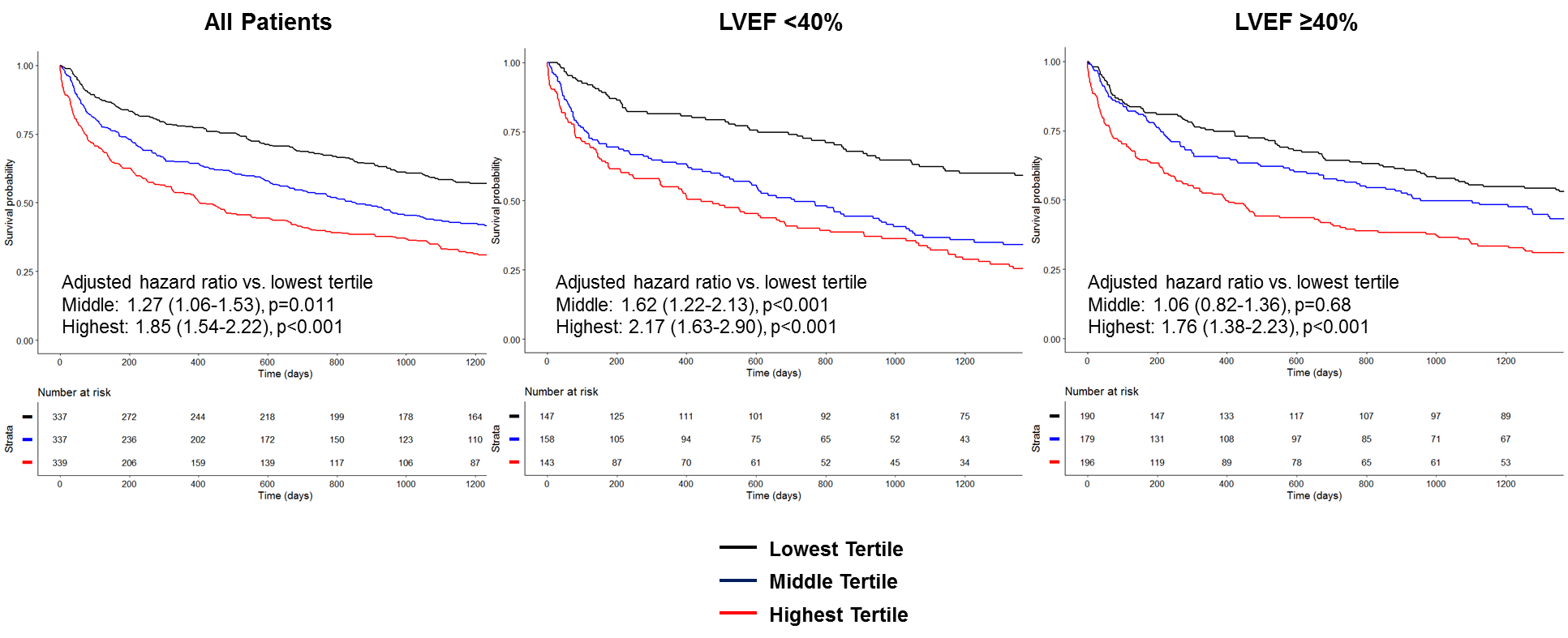
**

**Supplementary Table 1.** Summary of biomarkers reported in this study.

ADM - bioadrenomedullin

CA-125 = Cancer Antigen 125

CSTB = Cystatin-B

FGF-23 = Fibroblast growth factor 23

IL17RA = Interleukin-17 receptor A

IL18BP = Interleukin-18 binding protein

IL1RT1 = Interleukin-1 receptor type 1

IL1RT2 = Interleukin-1 receptor type 2

IL2RA = Interleukin-2 receptor subunit Alpha

IL6 = Interleukin-6

IL6RA = Interleukin-6 receptor subunit Alpha

MCP1 = Monocyte chemotactic protein 1

MPO = Myeloperoxidase

OPG = Osteoprotegerin

sST2 = Soluble suppression of tumorigenesis-2

TNFR1 = Tumor necrosis factor receptor 1

TNFR2 = Tumor necrosis factor receptor 2

TNFRSF14 = Tumor necrosis factor receptor superfamily member 14

TNFSF13B = Tumor necrosis factor ligand superfamily member 13B

TNRSF10C = Tumor necrosis factor receptor superfamily member 10C

UPA = Urokinase plasminogen activator

**Supplementary Table 2.** Association of NLR with outcomes in patients with unknown LVEF in the BIOSTAT-CHF cohort.

|  | **Hazard Ratio (95% CI)**  **(n=437)** | **p value** |
| --- | --- | --- |
| **Mortality and/or HF hospitalisation (153 events)** |  |  |
| Neutrophil count per SD increase | 1.22 (1.01-1.48) | **0.043** |
| Lymphocyte count per SD increase | 0.83 (0.65-1.06) | 0.14 |
| NLR per SD increase | 1.35 (1.16-1.58) | **<0.001** |
| NLR Tertile 1 | Baseline |  |
| NLR Tertile 2 | 1.05 (0.69-1.59) | 0.83 |
| NLR Tertile 3 | 1.20 (0.79-1.84) | 0.40 |
| **Mortality (97 events)** |  |  |
| Neutrophil count per SD increase | 1.36 (1.10-1.18) | **0.005** |
| Lymphocyte count per SD increase | 0.41 (0.28-0.60) | **<0.001** |
| NLR per SD increase | 1.60 (1.37-1.86) | **<0.001** |
| NLR Tertile 1 | Baseline |  |
| NLR Tertile 2 | 1.17 (0.66-2.10) | 0.59 |
| NLR Tertile 3 | 2.39 (1.38-4.13) | **0.002** |

All hazard ratios adjusted for the BIOSTAT-CHF risk model which includes age, HF hospitalisation in the previous year, peripheral oedema, systolic blood pressure, estimated glomerular filtration rate, urea, N‐terminal pro‐brain natriuretic peptide, haemoglobin, high‐density lipoprotein cholesterol, sodium, and beta‐blocker use.

Bold indicates p<0.05.

**Supplementary Table 3.** Association of NLR and biomarkers previously reported to be associated with outcome in BIOSTAT-CHF with outcomes in the BIOSTAT cohort adjusted for the BIOSTAT risk score and NT-proBNP.

|  | **Univariable Hazard Ratio (95% CI)** | **p value** | **Multivariable Hazard Ratio (95% CI)** | **p value** |
| --- | --- | --- | --- | --- |
| **Mortality and/or HF Hospitalisation** | | | | |
| NLR | 1.34 (1.28-1.42) | **<0.001** | 1.13 (1.04-1.20) | **0.002** |
| IL6 | 1.47 (1.38-1.56) | **<0.001** | 1.01 (0.92-1.11) | 0.82 |
| FGF-23 | 1.64 (1.54-1.76) | **<0.001** | 1.10 (1.01-1.21) | **0.036** |
| CA-125 | 1.56 (1.45-1.68) | **<0.001** | 1.07 (0.98-1.17) | 0.15 |
| Bioadrenomedullin | 2.01 (1.83-2.21) | **<0.001** | 1.24 (1.09-1.41) | **0.001** |
| **Mortality** |  |  |  |  |
| NLR | 1.40 (1.33-1.47) | **<0.001** | 1.14 (1.07-1.23) | **<0.001** |
| IL6 | 1.61 (1.50-1.73) | **<0.001** | 1.12 (1.02-1.24) | **0.016** |
| FGF-23 | 1.85 (1.72-2.00) | **<0.001** | 1.17 (1.05-1.30) | **0.003** |
| CA-125 | 1.72 (1.58-1.88) | **<0.001** | 1.17 (1.05-1.30) | **0.003** |
| Bioadrenomedullin | 2.58 (2.29-2.91) | **<0.001** | 1.63 (1.40-1.91) | **<0.001** |

Hazard ratios all per standard deviation increase in biomarker. Bold p<0.05.

Final multivariable model includes: BIOSTAT risk score, NT-proBNP, NLR, IL6, FGF-23, CA-125 and bioadrenomedullin.

**Supplementary Table 4.** Baseline characteristics of validation cohort (GoDARTS) by stratified by median NLR.

|  | **All patients**  **(N=1,013)** | | **NLR<4.29**  **(N=505)** | **NLR≥4.29**  **(N=508)** | **P value** |
| --- | --- | --- | --- | --- | --- |
| **Demographics** |  | | | | |
| Age (years) | 74 ± 10 | | 72 ± 11 | 75 ± 9 | **<0.001** |
| Female | 395 (39.0) | | 187 (37.0) | 208 (40.9) | 0.23 |
| Current Smoker | 327 (32.3) | | 168 (33.3) | 159 (31.3) | 0.55 |
| **Clinical Profile** |  | | | | |
| Systolic blood pressure (mmHg) | 141 ± 35 | | 140 ± 46 | 143 ± 44 | 0.28 |
| Diastolic blood pressure (mmHg) | 74 ± 7 | | 74 ± 7 | 73 ± 6 | 0.15 |
| **Echocardiographic Profile** |  | |  |  |  |
| LVEF≥40% | 565 (55.8) | | 289 (57.2) | 276 (54.3) | 0.38 |
| LVEF<40% | 448 (44.2) | | 216 (42.8) | 232 (45.7) |  |
| **Past medical history** |  | | | | |
| Myocardial infarction | 230 (22.7) | | 135 (26.7) | 95 (18.7) | **0.003** |
| Diabetes mellitus | 878 (86.7) | | 429 (85.0) | 449 (88.4) | 0.13 |
| **Medication** |  | | | | |
| ACEI/ARB | 607 (59.9) | 303 (60.0) | | 304 (59.8) | >0.99 |
| Beta-blockers | 596 (58.8) | 303 (60.0) | | 293 (57.7) | 0.49 |
| **Laboratory measurements** |  |  | |  |  |
| Total Cholesterol (mg/dl) | 4.6 ± 0.6 | 4.5 ± 0.6 | | 4.6 ± 0.5 | 0.17 |
| HDL Cholesterol (mg/dl) | 3.4 ± 3.3 | 3.4 ± 3.2 | | 3.6 ± 3.5 | 0.41 |
| Neutrophils (10^9^/L) | 7.2 ± 3.7 | 5.3 ± 2.0 | | 9.0 ± 4.0 | **<0.001** |
| Lymphocytes (10^9^/L) | 1.7 ± 2.3 | 2.1 ± 0.8 | | 1.1 ± 0.5 | **<0.001** |
