# Supplementary figures and images for: Correction to: ‘Neutrophil‐to‐lymphocyte ratio and outcomes in patients with new‐onset or worsening heart failure with reduced and preserved ejection fraction’
Source: ESC Heart Fail. 2023 Mar 31;10(3):2145. doi: 10.1002/ehf2.14362 (PMC10192285; doi:10.1002/ehf2.14362)

## Slide 1
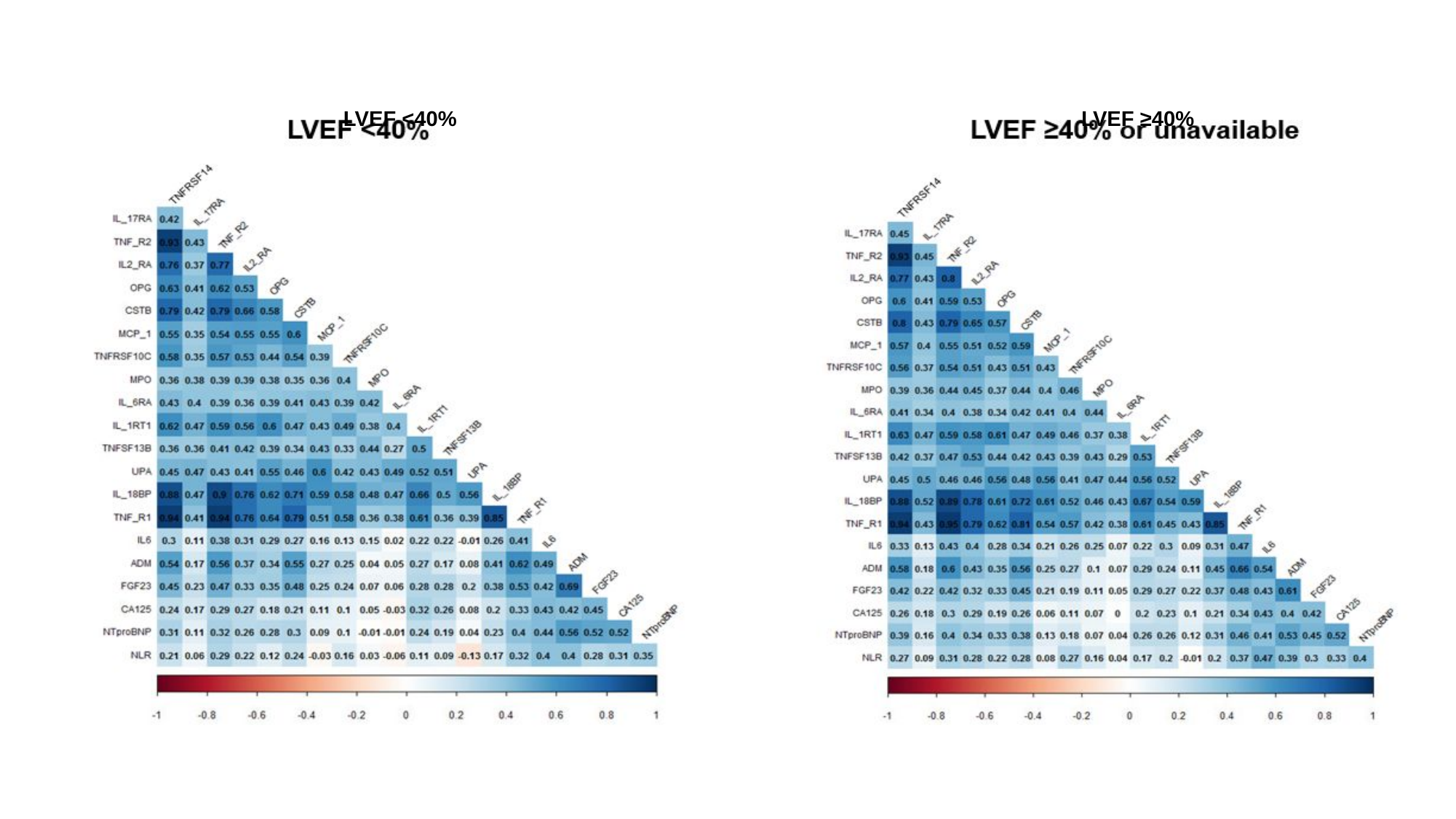

LVEF <40%
LVEF ≥40%

Supplement: Supplementary file 1 — Figure S1. Supporting Information. [file EHF2-10-2145-s001.pptx]
